# Did Ghana Do Enough? A Scientometric Analysis of COVID-19 Research Output from Ghana within the African Context

**DOI:** 10.3390/diseases11020056

**Published:** 2023-04-01

**Authors:** Akua K. Yalley, Selasie Ahiatrogah, Akuba B. Yalley, Isaac K. Yankson, Nicholas I. Nii-Trebi, Abena Asefuaba Yalley

**Affiliations:** 1 Department of Medical Laboratory Sciences, School of Biomedical and Allied Health Sciences, University of Ghana, Korle Bu, Accra P.O. Box 143, Ghana; 2 Department of Obstetrics and Gynaecology, College of Medicine, Pan African University of Life and Earth Sciences Institute, University of Ibadan, Ibadan P.O. Box 22133, Nigeria; 3 Department of Mining Engineering, University of Mines and Technology, Tarkwa P.O. Box 237, Ghana; 4 CSIR-Building and Road Research Institute, Kumasi P.O. Box UP40, KNUST, Ghana; 5 Zukunftskolleg, Department of Politics and Public Administration, University of Konstanz, 78464 Konstanz, Germany

**Keywords:** COVID-19, research, productivity, scientometric analysis, Ghana

## Abstract

The COVID-19 pandemic has generated worldwide research efforts to provide knowledge about the disease. Yet little is known about how Ghana contributed to this critical knowledge production. This scientometric analysis was conducted to ascertain Ghana’s COVID-19 research output within the African context to gain understanding and identify potential future directions. The study retrieved relevant research, spanning 2019 to 2022, from the Scopus database in December 2022. The retrieved data were assessed using various established indices, including collaboration patterns, productive institutions, citation patterns, and major research sponsors, among others. Ghana came seventh in Africa with a total of 1112 publications. For international collaborations, the United States and the United Kingdom were the major partners, while South Africa was the main African collaborator with Ghana. Out of the top 21 most productive authors, 85.7% were males and 14.3% were females, demonstrating a great gender gap in research output in Ghana. Although Ghana has made some contributions to the global COVID-19 research output, there are few intra-continental research collaborations, which limits Africa’s overall research output. Our study demonstrates a critical need for the Ghanaian government to prioritize research and funding and address barriers to women’s research productivity.

## 1. Introduction

SARS-CoV-2, a new betacoronavirus, was found to be the cause of a respiratory illness outbreak in December 2019 in Wuhan, China [1]. The Chinese Center for Disease Control and Prevention identified the causal agent from throat swab samples, and the World Health Organization (WHO) later named the infection coronavirus disease (COVID-19) [2]. Within a few weeks after its outbreak, the virus rapidly spread around the world. Whereas 80% of those that are infected develop mild-to-moderate symptoms that do not require hospitalization, less than 20% develop severe symptoms and become critically ill [3,4]. The first symptoms typically include shortness of breath, fever, and dry cough [2]. Other symptoms that have been associated with the illness include, but are not limited to, fatigue, anosmia, ageusia, expectoration, diarrhea, and pneumonia [5,6,7].

Since mid-February 2020, when the first COVID-19 case (confirmed using PCR testing) in Africa was reported, 57 African countries and territories have been afflicted by the pandemic. Globally, over 600-million cases of COVID-19 have been recorded. In addition, there were over 258,000 deaths due to COVID-19 in Africa by the end of December 2022 [8]. As of 31st December 2022, the total number of recorded COVID-19 cases in Ghana since March 2020 was 171,065, with 1461 COVID-19-related deaths [9]. Despite the fact that the disease was generally less severe in Ghana, it still had an impact on a number of areas, ranging from health to economic activities [10,11,12,13].

For many years, scientific publications have served as the primary means by which knowledge is communicated to both scientists as well as the general public. Thus, the rapid dissemination of information by scientists and stakeholders is essential for the timely implementation of measures to lessen the multiple effects of COVID-19 [14,15]. Kana et al. reiterated that prior to the increase in the occurrence of emerging illnesses in Africa, the continent’s contributions to the international output of scientific or peer reviewed publications may have been limited due to African institutions having inadequate investments in research and capacity building [16]. In fact, resource limitations in Africa and other developing countries do not only affect publication output, they also affect tools used in researching diseases and even tools for disease diagnostics [17]. This can have a negative impact on the quality of lives of citizens since significant issues specific to a particular area might take a while before coming to light. In recent times, health researchers in Africa are now positioned to be pivotal in clinical trials and, especially, in research on COVID-19 in Africa owing to international resources being deployed during disease outbreaks. Thus, the current pandemic may offer opportunities for enhancing research output in general. Like many other countries in the developing world, the pandemic significantly afforded the capacity for Africa to develop locally relevant knowledge and innovations [18,19]. Research organizations in Africa are in an advantageous position to enhance as well as maintain international collaborations in order to provide a more precise description of what is happening across the continent as the pandemic progresses.

As a developing nation, the challenges faced by Ghana when it comes to COVID-19 research are not much different from those faced in other kinds of research. Some of the challenges include limited funding and infrastructure, denial of access to research sites with potentially rich sources of information, and heightened bureaucracy when it comes to accessing samples and accompanying information relating to the topic of interest [20,21,22]. Other challenges include the lack of interest of potential study participants due to the failure to understand the positive impact the obtained data could bring to Ghana and, subsequently, the world at large. In addition, the lack of trust and lack of common values when it comes to collaboration [21,23] are challenges. In spite of the challenges and limited resources, researchers strive to work both locally and with international collaborators to publish their work. Yet little is known about the contributions of Ghanaian researchers to the global research output and the dynamics of COVID-19 research in Ghana. In times of a pandemic, a detailed assessment of a nation’s response and control efforts is crucial for informing global success. Scientific research publication output remains an essential denominator in measuring such a response. To that end, a scientometric analysis (which involves assessing the research effect by quantifying the publication output of a body of knowledge, taking into consideration the impact of research institutions, sponsorship sources, academic journals, and authors, among other parameters on the defined subject of study) constitutes a useful instrument for measuring a pandemic response in terms of the disease-related publication output [24]. This study employed a scientometric analysis technique to evaluate the contributions of Ghana to COVID-19 research within the African context between 2019 and 2022. This was done with the view to throw light on the research dynamics with respect to the COVID-19 pandemic, identify areas that need attention, and generate information that may feed into national policy on research aimed at controlling the pandemic.

## 2. Materials and Methods

Relevant data on COVID-19 and related research in Ghana and other most productive African countries (countries with the largest number of COVID-19 publications) from 2019–2022 were retrieved from Scopus International Database [http://www.scopus.com/search/ accessed on 28 December 2022] and analyzed. All data analyzed and reported here were based on what was retrieved as of 28th December 2022. Scopus was chosen because for peer reviewed literature, it is the largest citation and abstracting database, and it possesses tools that are suitable for the performance of scientometric analysis of this nature [25,26,27]. Scopus and Microsoft Excel were used for data analysis. Figures were generated in Scopus and Microsoft Excel (2016 version), and Tables were prepared in Microsoft Word (2016 version) based on information obtained from Scopus and Microsoft Excel analysis. For parameters with ranked results, automated analysis by scopus typically produced the top 15 results. Where ties made it necessary to go beyond 15, manual selection and analysis using Microsoft excel were performed.

### 2.1. Search Strategy for Most Productive Countries in Africa

For data on the most productive African countries in COVID-19 research, the search string below was used to first retrieve global data on COVID-19 research:

TITLE-ABS-KEY(coronavirus OR COVID-19 OR SARS-CoV-2) AND PUBYEAR > 2018 AND PUBYEAR < 2023

All countries retrieved were then ranked using an automated analysis option in Scopus per the number of COVID-19 publications. Then, the most productive African countries were selected by eliminating non-African countries. In addition, the dataset on the most productive African countries was confirmed by a separate advance query string with specific African countries. For example, data on Nigeria were retrieved using the string below:

(TITLE-ABS-KEY (coronavirus OR COVID-19 OR SARS-CoV-2) AND AFFIL(Nigeria)) AND PUBYEAR > 2018 AND PUBYEAR < 2023

### 2.2. Search Strategy for COVID-19 Data on Ghana

An advanced search strategy that involved “coronavirus” OR “COVID-19” OR “SARS-CoV-2” as the keywords in the “Title”, “Abstract”, and “Keywords” fields together with “Ghana” in the “affiliation field” and limiting the data range from 2019 to 2022 was used for the purpose of searching for data on Ghana. The exact search strategy that was used in the advanced search in order to extract data about Ghana is as presented in the format below:

(TITLE-ABS-KEY(coronavirus OR COVID-19 OR SARS-CoV-2) AND AFFIL(Ghana)) AND PUBYEAR > 2018 AND PUBYEAR < 2023

The primary search string results also provided information on international collaborations, publication types, most productive institutions, the most productive authors, journals, and funding sources, among others that were used for analysis.

### 2.3. Search Strategy for Various Subject Areas Related to Ghana COVID-19 Research

With regards to data retrieval and analyses of various subject areas related to Ghana, the following search strategy was restricted to specific topics, such as the advanced search string below to collect data on medicine:

(TITLE-ABS-KEY (coronavirus OR COVID-19 OR SARS-CoV-2) AND AFFIL (Ghana)) AND PUBYEAR > 2018 AND PUBYEAR < 2023 AND (LIMIT-TO (SUBJAREA,”MEDI”))

### 2.4. Strategy for Obtaining Data on Citations

To obtain data on citations, the main search string about Ghana was run to collate publications. Then, the “citation” tag was checked, which rearranged the output in descending order so that the publications with the most citations were at the top. These publications were then marked and assessed for further analysis.

## 3. Results

### 3.1. Africa’s Most Productive Countries and Where Ghana Lies with Regards to COVID-19 Research Output

**Figure 1:**
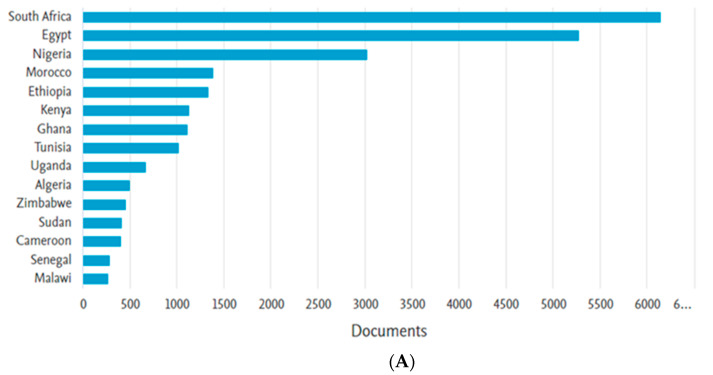

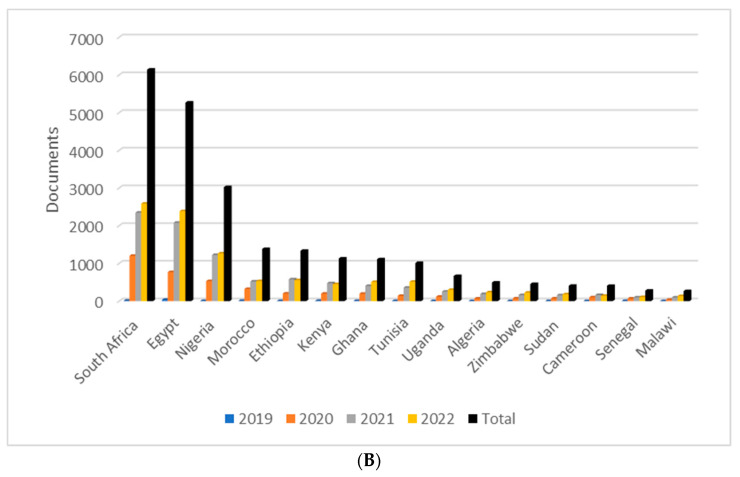
Africa’s COVID-19 Research Output: The Most Productive Countries in Africa (**A**): The top 15 most productive countries in Africa from 2019 to 2022 presented in order of cumulative number of publications; (**B**): Figure displays both cumulative number of publications as well as publications by year over the study period for the top 15 African countries.

In order to determine the general COVID-19 output in Africa in relation to where Ghana’s own input lay, we searched for the number of publications coming out of Africa in general and ranked the most productive 15 countries. Figure 1A shows the total publications by the most productive African countries from 2019 to 2022. South Africa made the most publications among the top 15 countries in Africa, with 6142 publications. Egypt ranked second (5268 publications), followed by Nigeria (3023), Morocco (1382), Ethiopia (1334), and Kenya (1130). Ghana was ranked seventh among the top 15 countries with 1112 publications. The lowest among the top 15 was Malawi, which contributed a total of 266 publications over the period. Considering individual years, Ghana maintained a constant position as the seventh-most productive nation in the three successive years from 2020 to 2022, with 196 publications, 405 publications, and 506 publications, respectively. However, in 2019, Ghana was the fifth-most productive country with five publications.

Generally, with the exception of Ethiopia, which has a publication output that decreased marginally between 2021 and 2022 (from 577 to 553), all the other countries showed a steady increase in terms of their research outputs over the period examined (Figure 1B).

Of note, out of the top 15 performing countries, five out of the seven countries from North Africa (Egypt, Morocco, Tunisia, Algeria, and Sudan) were represented; five out of the 22 Eastern African countries (Ethiopia, Kenya, Uganda, Zimbabwe, and Malawi) were represented; three out of the 17 countries from West Africa (Nigeria, Ghana, and Senegal) were represented; one out of the nine countries from Central Africa was represented (Cameroon); and one out of the five Southern African countries (South Africa) was represented.

### 3.2. Ghana’s COVID-19 Research Output from 2019–2022: Published Document Types

**Figure 2:**
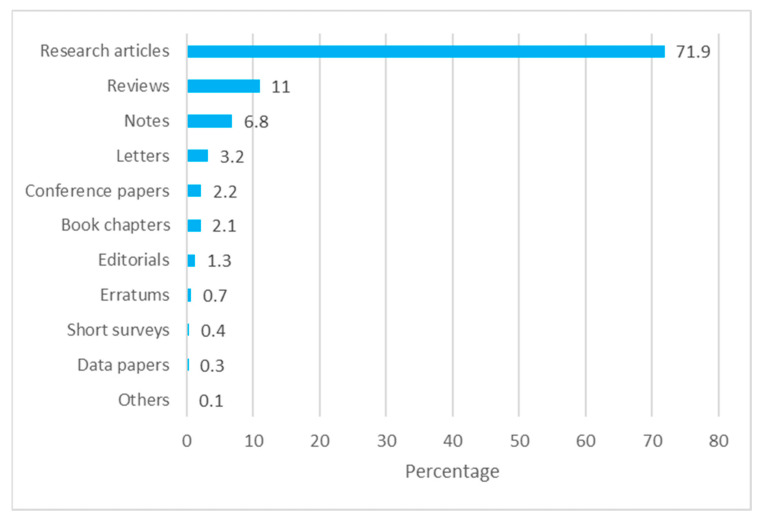
Published COVID-19 documents by type from Ghana.

Of the 1112 publications from Ghana, the majority (71.9%) were research articles, followed by reviews that formed 11% of the total publications during the period from 2019–2022. Notes, letters, and conference papers represented 6.8%, 3.2%, and 2.2% of the total publications, respectively. Editorials represented 1.3%. The rest were below 1% each of COVID-19 publications. These data are represented in Figure 2.

### 3.3. International Collaborations in Ghana’s COVID-19 Publication Output from 2019–2022

From 2019–2022, a large number of publications from Ghana had international collaborators, with some papers having collaborators from multiple countries. The United States was Ghana’s major international partner in research, resulting in 296 publications and representing 26.62% of Ghana’s total publication output. In all, out of 16 international collaborations, 12 were with nations and institutions outside Africa, with only four being with African nations: South Africa, Kenya, Nigeria, and Cameroon. Overall, Ghana’s international collaboration was appreciably diverse, involving Europe, Asia, and the Americas, among others. Details of these international collaborations are shown in Table 1.

### 3.4. Ghana’s Publication Output by Subject Area

**Figure 3:**
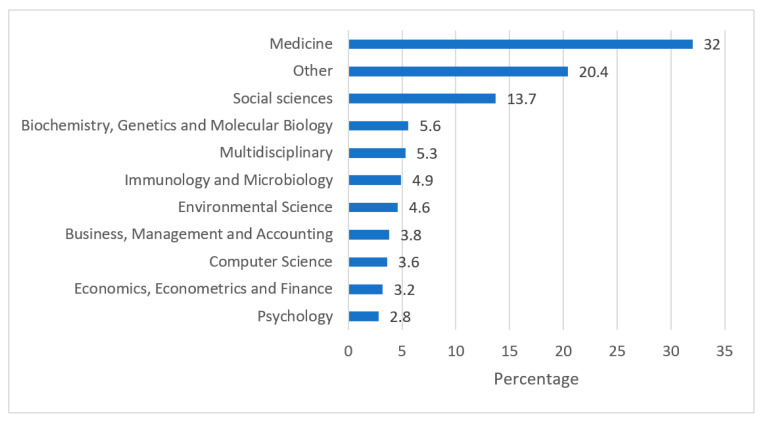
Ghana’s COVID-19 research output by subject area from 2019 to 2022.

Ghana’s publication output in COVID-19 research from 2019–2022 occurred in the context of more than 10 subject areas, as shown in Figure 3 (according to subject-based database categorizations of scholarly journals). “Medicine” had the highest output with 32%, and “Social Sciences” had a 13.7% output. “Other” subject areas encompassing pharmacology, toxicology and pharmaceutics, nursing, and veterinary, among others, collectively represented 20.4%.

### 3.5. Most Productive Ghanaian Institutions in COVID-19 Research from 2019–2022

**Figure 4:**
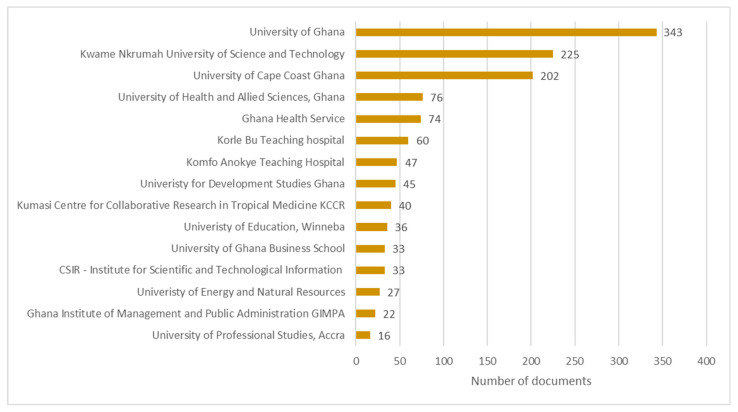
Most productive Ghanaian institutions publishing COVID-19 research from 2019 to 2022.

The top 15 Ghanaian institutions involved in COVID-19 research from 2019–2022, as shown in Figure 4, had an average of 85 publications per institution.

Three of these institutions contributed more publications than the average number. Incidentally, these are the three oldest public universities. The University of Ghana topped the list with 343 publications, followed by the Kwame Nkrumah University of Science and Technology with 225 publications, while the University of Cape Coast was next with 202 publications. Ten of the top 15 most productive institutions were academic in nature; two were teaching hospitals; two were research institutions; and one was a government body responsible for administering government-provided health services and healthcare policies.

### 3.6. Characteristics of the Most Productive Ghanaian Researchers on COVID-19 Research

The top 21 most productive authors published at least 10 or more COVID-19 publications between 2019 and 2022. These authors together contributed 266 publications, with an average of 12.7 publications per author, and accounted for 23.9% of publications on COVID-19 research output from 2019–2022. The first five authors included Amuasi, J.H. (20 publications), Hagan, J.E. (19 publications), Owusu, M. (19 publications), Sam-Agudu, N.A. (18 publications), and Ahinkorah, B.O. (15 publications). Adam, A.M., Ameyaw, E.K., Ayanore, M.A., and Seidu, A.A. each produced 13 COVID-19 publications. These were followed by Asafo-Adjei, E., Phillips, R.O., and Vanderpuye, V., who authored 11 publications each. In addition, Abu, E.K., Adu-Sarkodie Y., El-Duah, P., Kenu, E., Owusu Junior, P., Quansah, F., Marcarious, M. M., Yeboah, R., and Yeboah-Manu, D. each contributed 10 publications to Ghana’s COVID-19 research output. Among the top 21 most productive authors, 17, representing 85.7%, were males, while three, representing 14.3%, were females with the most productive female (Sam-Agudu, N. A.) being the fourth-most productive author in Ghana. Table 2 summarizes the characteristics of the top 21 most prolific authors.

### 3.7. Journals Publishing the Highest Number of Ghana’s COVID-19 Documents from 2019–2022

The top 15 journals (local and foreign) publishing Ghanaian research papers from 2019 to 2022 collectively produced 250 COVID-19 publications. This accounts for 22.48% of the total output from Ghana. Among the journals with the most published articles, Plos One published the highest number of COVID-19 documents with 41 publications, followed by Ghana Medical Journal and Pan African Medical journal with 36 publications each. The journal Frontiers in Public Health produced 18 publications, followed by the International Journal of Environmental Research and Public Health with 17. The rest had fewer than 15 publications, with the last journals, BMJ Open, Complexity, and Nature Medicine, having eight publications each. Out of the 15 journals, 13 were open access. In addition, only one journal, Ghana Medical Journal, was a Ghanaian-based journal. These findings are presented in Table 3.

### 3.8. Characteristics of Highly-Cited Papers from 2019 to 2022

The overall number of citations of publications with the participation of Ghanaian authors was 9330. Of the top 15 highly cited publications, 13 were international collaborative publications, and two were national collaborations. The citation of these publications ranged from 84 to 421. Overall, these publications contributed to 2514 citations, which represents 26.9% of the overall citation of Ghana’s COVID-19 research output from 2019–2022, with the average being 167.6 citations. Out of the 15 highly cited publications, 10 were research articles, three were reviews, one was an editorial, and one was a note. Based on the subject-based database categorizations of the journals in which these publications were made, a majority (10 out of the 15) were in the context of medicine, with a total of 1940 citations. Two were published in journals belonging to medicine as well as immunology and microbiology subject areas, with a total of 326 citations. One was in the genetics, biochemistry, and molecular biology subject areas, with a total of 189 citations. In addition, one was published in the health professions of toxicology, pharmacology, and pharmaceutics subject areas, with a total of 113 citations. Other subject areas that were featured in the most highly cited publications included one publication from the mathematics, astronomy, and physics subject areas (94 citations); one from a multidisciplinary subject area (94 citations); and one from environmental science. Social science and decision sciences subject areas included 84 citations. The most highly cited paper was in the subject category medicine, specifically pediatrics, perinatology, and child health. Among the top 15 highly cited publications, four publications had Ghanaian authors as the first author. These authors were Ayittey, F. K. with 236 citations; Kretchy, I. A. with 113 citations; Asamoah, J. K. K. with 94 citations; and Dzisi, E. K. Jr. with 84 citations. Other characteristics can be found in Table 4.

### 3.9. Top Funding Sources for Ghana’s COVID-19 Research Output from 2019–2022

**Figure 5:**
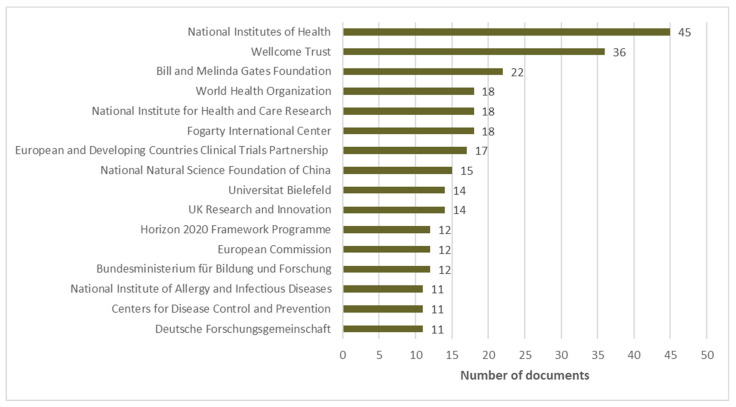
Top 16 sponsors of Ghana’s COVID-19 research output.

International organizations were the leading funding sources for COVID-19 research in Ghana from 2019–2022 as shown in Figure 5.

The top 16 sponsors of COVID-19/SARS-Cov-2 research in Ghana together funded 274 publications, representing 24.6% of the total research productivity and an average of 18 publications per sponsor. Three of the sponsors funded a higher number of research than the group average. These sponsors include the National Institutes of Health, Wellcome Trust, and the Bill and Melinda Gates Foundation which funded 45, 36, and 22 publications, respectively. Other sponsors include Fogarty International Center, the National Institute for Health and Care Research, and The World Health Organization, with each funding 18 publications. The European and Developing Countries Clinical Trials Partnership funded 17 publications, while the National Natural Science Foundation of China also sponsored 15 COVID-19 research in Ghana. The rest of the sponsors had fewer than 15 publications, with the last three sponsors providing funding for 11 publications each.

## 4. Discussion

Due to the persistent COVID-19 pandemic, there has been a significant increase in efforts to identify effective methods for prevention and treatment in order to lessen the impact of the virus. The analysis of this scientometric study established that all forms of research on COVID-19 were being conducted in Ghana and that the COVID-19 research output also varied in the African region. As reported by Edem et al., despite Africa’s size and population, it holds comparatively fewer COVID-19 trials than other regions [43]. Although experiencing relatively lower morbidity and mortality rates from the COVID-19 pandemic compared to other regions [44], Africa has still made appreciable contributions to global research on the virus. According to this study, South Africa had the highest number of COVID-19 research publications in Africa, with a total of 6142 publications. This result is consistent with previous studies that found South Africa to have the most prolific output of health research on the continent [16,45,46]. Another study, not related to the coronavirus disease, stated that only three countries in Africa (Egypt, South Africa, and Nigeria) provide about half of the total research funding on the continent [47]. Previous research established that a country’s Gross Domestic Product (GDP) is a key factor in determining research capacity [46]. These findings are corroborated by our study, which showed that South Africa, Egypt, and Nigeria, which have the highest GDPs in Africa [48], also were the most productive countries in COVID-19 research on the continent from 2019 to 2022.

As of March 2020, Ghana was among the last African countries to have recorded the coronavirus disease and lagged slightly behind other countries in the region and significantly behind the rest of the world, especially during the second wave of the pandemic [49]. In this study, Ghana occupied the seventh position in Africa with a research output of 1112 publications, which represents an appreciable contribution to the continent’s total COVID-19 research output from 2019–2022. The outbreak of COVID-19 led to new opportunities for research collaborations and facilitated the creation of international partnerships for academic exchanges and joint research efforts [50]. The United States and the United Kingdom were the top collaborators on COVID-19 research in Ghana, contributing a high volume of publications to Ghana’s output. Regarding collaborations with other African countries, South Africa was the top collaborator on COVID-19 research for Ghana, making South Africa a major reference for research collaboration on COVID-19 in Africa and globally. The distribution of Ghanaian COVID-19 research output indicated that 32% of the publications were in the medicine subject area. It was anticipated that the medical field would have the highest number of publications among all sub-categories due to the high infection and mortality rates of the disease, making it crucial to understand the disease progression. However, social sciences also had a significant number of publications, accounting for 13.7%, which may be attributed to the social consequences of the pandemic, including the unprecedented changes in lifestyle, work, and social interactions that affected human relations due to the disease [51].

Among the most productive Ghanaian authors, the majority were affiliated with Ghanaian institutions, while a relatively small number was affiliated with non-Ghanaian institutions. Of the top 21 most productive Ghanaian authors, 85.7% were male and 14.3% were female, demonstrating a great gender gap in research contribution and output in Ghana. While this could be attributed to the general significant gender disparity in science, technology, engineering, and mathematics (STEM) fields in Ghana as well as cultural practices that can affect women’s ability to excel academically [52,53,54], our study points to the concern of less female representation in research output across all disciplines. However, it was still encouraging to find females among the top 21, with the most productive female occupying the fourth place. It is also worthy of note that the second-most cited Ghanaian first author was female. Our analysis showed that only two African journals were among the most productive, with the Ghana Medical Journal and Pan African Medical Journal both having 36 publications each. However, the majority of Ghanaian COVID-19 research output was published in foreign journals. Previous research has indicated that medical professionals often prioritize publishing in foreign journals over local ones due to the wider readership of foreign journals [55]. Our study found that most of the top-cited articles were published in high-impact journals. Most of these articles were published in 2020 and 2021, making them among the earliest sources of scientific knowledge on the pandemic and likely to be heavily relied upon by other scientists, resulting in high citation rates. However, it was noted that only a small number of the most cited articles appeared on the list of the top 15 journals, with the highest number of published documents. The first most cited article was by Villar et al., 2021, and was published in JAMA Pediatrics. The aforementioned study was a multinational cohort study that focused on maternal and neonatal mortality rates among pregnant women and involved 50 authors from countries across Africa, the United States, and Europe, which may have contributed to its high citation rate. In addition, our study revealed that the United States, United Kingdom, European Union, and China are the leading sources of funding for Ghana’s COVID-19 research output, which may be due to the high prevalence of COVID-19 in these countries during the initial outbreak [44] and also having the financial capacity to fund multiple research projects and prioritizing research. To increase Ghana’s overall COVID-19 research output, the Ghana government and other local funding sources should make research a priority and provide more funding to strengthen capabilities at all levels to enhance not only COVID-19 and health related research, but also research in other areas in Ghana.

A limitation of this study is that only the Scopus database was used for data retrieval. While other databases may contain COVID-19 publications not included in the Scopus database, we based our study on high-quality data and comprehensive records from Scopus, and the data retrieved can give a fair idea as far as research productivity output from a region is concerned. In addition, the number of citations per article and total citations may vary with time, hence that measure is often disputed as a complete indicator of the quality of an article or author. Likewise, the number of publications is not the only indicator of a journal’s impact or its productivity. Other metrics, including impact factor, Citespace, and SCImago Journal Rank (SJR) indicator, may be used in future studies. In addition, even though international collaborative research is prominent in Ghana, it is difficult to clearly indicate the level of contribution of each country in an international collaborative paper. Additionally, since this study relied solely on Scopus analysis, with specific numbers retrieved on 28th December 2022, database updates may result in discrepancies. However, we believe that the data retrieved from 2019 to 2022 captured the crucial evolution of COVID-19, including the initial outbreak, the second wave of the pandemic, and vaccine development and administration. There is also the possibility that the results could include studies on older types of coronaviruses. However, this would be minimal since the period studied was at the peak of the COVID-19 pandemic. Hence, most coronavirus studies focused on COVID-19.

## 5. Conclusions

Although the impact of COVID-19 in Africa as compared to other regions of the world has been less severe, Africa and Ghana have contributed fairly well to the global COVID-19 research output. However, collaborations and partnerships should be encouraged among African countries to increase Africa’s research output. Funding organizations in Ghana and the government should be encouraged to prioritize and fund research. In addition, capacity building should be promoted among Ghanaian female authors to increase their productivity and Ghana’s cumulative research output.

## Figures and Tables

**Table 1:** The top sixteen International Collaborations in Ghana’s COVID-19 Publication output from 2019 to 2022.

Rank	Countries	Number of Joint Publications (%)
1st	United States	296 (26.6%)
2nd	United Kingdom	279 (25.1%)
3rd	South Africa	194 (17.5%)
4th	Nigeria	174 (15.7%)
5th	Germany	117 (10.5%)
6th	Australia	113 (10.2%)
7th	India	110 (9.9%)
8th	Canada	106 (9.5%)
9th	Kenya	92 (8.3%)
10th	China	89 (8.0%)
11th	Switzerland	73 (6.6%)
12th	Brazil	70 (6.3%)
13th	Pakistan	58 (5.2%)
14th	Cameroon	53 (4.8%)
14th	Italy	53 (4.8%)
14th	Malaysia	53 (4.8%)

**Table 2:** Characteristics of the top 21 most productive Ghanaian researchers on COVID-19 from 2019 to 2022.

Rank	Author	Sex	Affiliation	No. of Pub.
1st	Amuasi, J. H.	Male	Kwame Nkrumah University of Science and Technology, Kumasi, Ghana	20
2nd	Hagan, J. E.	Male	University of Cape Coast, Cape Coast, Ghana	19
2nd	Owusu, M.	Male	Kwame Nkrumah University of Science and Technology, Kumasi, Ghana	19
3rd	Sam-Agudu, N. A.	Female	University of Cape Coast, Cape Coast, Ghana	18
4 th	Ahinkorah, B. O.	Male	University of Technology, Sidney, Australia	15
5 th	Adam, A. M.	Male	University of Cape Coast, Cape Coast, Ghana	13
5 th	Ameyaw, E. K.	Male	Lingnan University, Hong Kong, Hong Kong,	13
5 th	Ayanore, M. A.	Male	University of Health and Allied Sciences, Ho, Ghana	13
5 th	Seidu, A. A.	Male	University of Cape Coast, Cape Coast, Ghana	13
6 th	Asafo-Adjei, E.	Male	University of Cape Coast, Cape Coast, Ghana	11
6th	Phillips, R. O.	Male	Kwame Nkrumah University of Science and Technology, Kumasi, Ghana	11
6th	Vanderpuye, V.	Female	Korle Bu Teaching Hospital, Accra, Ghana	11
7th	Abu, E. K.	Male	University of Cape Coast, Cape Coast, Ghana	10
7th	Adu-Sarkodie, Y.	Male	Kwame Nkrumah University of Science and Technology, Kumasi, Ghana	10
7th	El-Duah, P.	Male	Charité—Universitätsmedizin Berlin	10
7th	Kenu, E.	Male	University of Ghana, Accra, Ghana	10
7th	Owusu Junior, P.	Male	University of Cape Coast, Cape Coast, Ghana	10
7th	Quansah, Frank	Male	University of Education, Winneba, Ghana	10
7th	Marcarious M. T.	Male	Tehran University of Medical Science/University of Ghana	10
7th	Yeboah R.	Male	Kumasi Centre for Collaborative Research in Tropical Medicine (KCCR), Kumasi, Ghana	10
7th	Yeboah-Manu, D.	Female	University of Ghana, Accra, Ghana	10

**Table 3:** Top 15 most productive journals publishing Ghana’s COVID-19 research output from 2019 to 2022.

Rank	Journal	No. of Publications	Journal Characteristics
1st	Plos One	41	Open access
2nd	Ghana Medical Journal	36	Open access
2nd	Pan African Medical Journal	36	Open access
3rd	Frontiers In Public Health	18	Open access
4th	International Journal of Environmental Research And Public Health	17	Open access
5th	BMJ Global Health	14	Open access
5th	Lancet	14	Hybrid
6th	Scientific African	11	Open access
7th	Heliyon	10	Open access
7th	International Journal Of Infectious Diseases	10	Open access
7th	Lancet Global Health	10	Open access
8th	BMC Public Health	9	Open access
9th	BMJ Open	8	Open access
9th	Complexity	8	Open access
9th	Nature Medicine	8	Hybrid

**Table 4:** Characteristics of top 15 highly cited COVID-19 publications from 2019 to 2022.

Rank	Authors	Document Title	Journal Title/Subject Area of Journal	Citation	Publication Type
1st	Villar et al., 2021 [28]	Maternal and Neonatal Morbidity and Mortality among Pregnant Women with and without COVID-19 Infection: The INTERCOVID Multinational Cohort Study	JAMA Pediatrics/Medicine	421	Research article
2nd	Lamontagne et al., 2020 [29]	A living WHO guideline on drugs for COVID-19	The BMJ/Medicine	416	Research article
3rd	Ayittey et al., 2020 [30]	Economic impacts of Wuhan 2019-nCoV on China and the world	Journal of Medical Virology/Medicine; Immunology and Microbiology	236	Note
4th	Chakaya et al., 2021 [31]	Global Tuberculosis Report 2020—Reflections on the Global TB burden, treatment and prevention efforts	International Journal of Infectious Diseases/Medicine	216	Research article
5th	Kontis et al., 2020 [32]	Magnitude, demographics and dynamics of the effect of the first wave of the COVID-19 pandemic on all-cause mortality in 21 industrialized countries	Nature Medicine/Biochemistry, Genetics and Molecular Biology	189	Research article
6th	Tabah et al., 2020 [33]	Personal protective equipment and intensive care unit healthcare worker safety in the COVID-19 era (PPE-SAFE): An international survey	Journal of Critical Care/Medicine	137	Research article
7th	Zhou et al., 2021 [34]	Global epidemiology, health burden and effective interventions for elevated blood pressure and hypertension	Nature Reviews Cardiology/Medicine	135	Review
8th	Kretchy et al., 2021 [35]	Medication management and adherence during the COVID-19 pandemic: Perspectives and experiences from low-and middle-income countries	Research in Social and Administrative Pharmacy/Health Professions; Pharmacology, Toxicology and Pharmaceutics	113	Research article
9th	Haider et al., 2020 [36]	Lockdown measures in response to COVID-19 in nine sub-Saharan African countries	BMJ Global Health/ Medicine	109	Review
10th	Paulson et al., 2021 [37]	Global, regional, and national progress towards Sustainable Development Goal 3.2 for neonatal and child health: all-cause and cause-specific mortality findings from the Global Burden of Disease Study 2019	The Lancet/Medicine	94	Research article
10th	Asamoah et al., 2020 [38]	Global stability and cost-effectiveness analysis of COVID-19 considering the impact of the environment: using data from Ghana	Chaos, Solitons and Fractals/Mathematics; Physics and Astronomy	94	Research article
10th	Sanchez-Felipe et al., 2021 [39]	A single-dose live-attenuated YF17D-vectored SARS-CoV-2 vaccine candidate	Nature/Multidisciplinary	94	Research article
11th	Abena et al., 2020 [40]	Chloroquine and hydroxychloroquine for the prevention or treatment of COVID-19 in Africa: Caution for inappropriate off-label use in healthcare settings	American Journal of Tropical Medicine and Hygiene/Medicine; Immunology and Microbiology	90	Review
12th	Petersen et al., 2022 [41]	Emergence of new SARS-CoV-2 Variant of Concern Omicron (B.1.1.529)—highlights Africa’s research capabilities, but exposes major knowledge gaps, inequities of vaccine distribution, inadequacies in global COVID-19 response and control efforts	International Journal of Infectious Diseases/Medicine	86	Editorial
13th	Dzisi et al., 2020 [42]	Adherence to social distancing and wearing of masks within public transportation during the COVID 19 pandemic	Transportation Research Interdisciplinary Perspectives/Social Sciences; Decision Sciences; Environmental Science	84	Research article

## Data Availability

Data generated and presented in this study are available on request from the corresponding authors.
